# A prospective, observational study on conversion of clinically isolated syndrome to multiple sclerosis during 4-year period (MS NEO study) in Taiwan

**DOI:** 10.1371/journal.pone.0202453

**Published:** 2019-07-15

**Authors:** Long-Sun Ro, Chih-Chao Yang, Rong-Kuo Lyu, Kon-Ping Lin, Tzung-Chang Tsai, Shiang-Ru Lu, Kuo-Hsuan Chang, Li-Chieh Huang, Ching-Piao Tsai

**Affiliations:** 1 Department of Neurology, Chang Gung Memorial Hospital-Linkou Medical Center, Chang Gung University College of Medicine, Taoyuan, Taiwan, R.O.C.; 2 Division of Nephrology, Department of Internal Medicine, Department of Internal Medicine, Kaohsiung Chang Gung Memorial Hospital and Chang Gung University College of Medicine, Kaohsiung, Taiwan, R.O.C.; 3 Department of Neurology, Neurological Institute, Taipei Veterans General Hospital and National Yang-Ming University, Taipei, Taiwan, R.O.C.; 4 Department of Neurology, Taichung Hospital, Ministry of Health and Welfare, Taichung, Taiwan, R.O.C.; 5 Department of Neurology, Kaohsiung Medical University Hospital, Taiwan, R.O.C.; 6 Merck Biopharma (Taiwan), Taipei, Taiwan, R.O.C.; 7 Department of Biotechnology, Asia University, Taichung, Taiwan, Taipei Beito Health Management Hospital, Taipei, Taiwan, R.O.C.; Heinrich-Heine-Universitat Dusseldorf, GERMANY

## Abstract

**Importance:**

CIS to MS conversion rates vary depending on population cohorts, initial manifestations, and durations of follow-up.

**Objective:**

To investigate conversion rate of patients from CIS to MS and the prognostic significance of demographic and clinical variables in Taiwanese population.

**Design:**

Nationwide, prospective, multi-centric, observational study from November 2008 to November 2014 with 4 years follow-up.

**Setting:**

Multi-centre setting at 5 institutions in Taiwan.

**Participants:**

152 patients having single clinical event potentially suggestive of MS in last 2 years were enrolled as consecutive sample. 33 patients were lost to follow-up and 16 patients did not complete the study.103 patients completed the study.

**Intervention(s) (for clinical trials) or exposure(s) (for observational studies):**

Natural progression from first episode of CIS to MS or NMO was observed.

**Main outcome(s) and measure(s):**

Variables analysed were ‘proportion of patients converting to MS or NMO after first episode of CIS’, ‘duration between first episode of neurological event and diagnosis of MS’, ‘status of anti-AQP4 IgG’ and ‘length of longest contiguous spinal cord lesion in MS patients’. Association between baseline characteristics and progression to MS from CIS was analyzed using multiple logistic regression. Multivariate time dependent effect of baseline characteristics on progression to MS was plotted.

**Results:**

14.5% patients with CIS converted to MS after 1.1 ± 1.0 years with greater predisposition (18.8%) in those having syndromes referable to the cerebral hemispheres. Conversion rate from ON to MS was 9.7%. 90.9% patients had mild disease course. 46.7% patients had abnormal MRIs at baseline, with 0.6±0.5 contrast enhanced lesions. ‘Below normal BMI’ and ‘MRI lesion load (≥ 4 lesions)’ were identified as risk indicators for the development of MS. Amongst the patients who developed NMO as diagnosed by modern criteria, 80% were positive for anti-AQP4 IgG antibody.

**Conclusions and relevance:**

‘Below normal BMI’ and ‘number of demyelinating lesions (≥4)’ are significant predictors of conversion from CIS to MS. A low conversion rate to MS in Taiwanese CIS patients and majority of them having a mild course and minimal disability suggest the roles of geographic, genetic and ethnic factors.

**Trial registration:**

Non-trial observational study.

## Introduction

Clinically isolated syndrome (CIS) is the first symptomatic neurologic episode caused by demyelination in the central nervous system (CNS). It may or may not be a precursor of multiple sclerosis (MS). The conversion rate from CIS to MS ranges from 30% to 82% [1,2,3,4,5]. None of the studies has prospectively analysed the conversion of CIS to MS in Taiwanese population. The wide variability in conversion rates is attributable to geographical area, ethnic group, initial manifestation, and the duration of follow-up. Other factors like difference in patient selection method, diagnostic criteria, and study design may also cause variability.

CIS is a stage of diagnostic and therapeutic dilemma because a possible intervention could reduce the chances of conversion to MS. Prognostic factors regarding the conversion to MS may help to screen patients and expedite treatment. [6,7]. Numerous prognostic factors for conversion to MS after the first episode of CIS have been identified. Of the epidemiological risk factors, ‘female gender’ and ‘young age’ are predictive of conversion to MS [2–5,8–10]. Long term studies have shown that ‘number and activity of MRI lesions’ and ‘spinal cord abnormalities at the time of presentation’ are strong predictors of conversion to MS [7,11]. Regarding location, Barkhof *et al*. suggested that ‘periventricular demyelinating lesions’ foresee conversion to MS [12]. A ‘shorter interval to a second episode’ and the ‘number of relapses in the first 2 years’ are also predictors of a poor prognosis [13].

Optic neuritis, an inflammatory demyelination of the optic nerve, is the clinical phenotype of CIS in 25% patients. [1,14]. In Taiwan, a retrospective single-centre study revealed that conversion rate of ON to MS was 14.3%. ‘Female gender’, ‘retro bulbar ON’, ‘MRI abnormalities’, ‘elevated CSF IgG index’, and ‘recurrent attacks’ were identified as risk factors [2]. Another Taiwanese study reported the cumulative incidence of ON from 2001 to 2004 to be 1.33 per 1,000 persons. The cumulative incidence of MS from new diagnosis of ON was 0.78% between 2001 and 2004. [15]

Neuromyelitis optica (NMO) is a differential diagnosis of CIS [1]. Many patients with NMO have detectable serum IgG antibodies against the water channel aquaporin-4 (AQP4–immunoglobulin G [IgG]), specific to NMO. However, in 20–30% of patients with NMOSD, AQP4 IgG Abs are not detectable depending on the assay used. Many studies have shown presence of serum-Abs against myelin-oligodendrocyte-glycoprotein (MOG) in AQP4 IgG Ab negative NMOSD patients. [16,17]

Additionally, longitudinally extensive spinal cord lesions are also suggestive of NMO [1]. Based on this finding, the revision of NMOSD diagnostic criteria in 2015 [18] included disorders with MOG-Abs in the NMO spectrum, with or without evidence of anti-AQP4 IgG. In the latest 2017 revisions of the McDonald criteria also [19], the International Panel on the Diagnosis of MS has recommended that testing for anti-AQP4 and anti-MOG antibodies should be performed, when available, in patients with features of NMOSD. However, presence of anti-MOG antibody could not be tested in this study since it was conceptualized and conducted prior to the role anti-MOG antibody in NMOSD diagnosis was known.

MS is diagnosed mainly by clinical acumen supported by investigations, including T2-weighted magnetic resonance imaging (MRI), CSF evaluation, and visual evoked potential [19]. MRI remains the most important surrogate marker for predicting the risk of a second event in CIS patients (i.e. conversion to MS) but the clinical outcome remains unpredictable due to the high variability. The prediction of occurrence of further neurological events and conversion to MS is essential both for patients and neurologists and hence, the search for auxiliary markers that could provide additional information about the disease course, must continue.

This study was planned with the primary objective of evaluating the conversion rate from CIS to MS over a 4-year period in Taiwanese patients. The secondary objectives were to assess relationship between CIS and MS in terms of duration between first episode of neurological event to diagnosis of MS, proportion of patients with conventional MS or NMO, and association between baseline demographics/disease characteristics and progression to MS. The other secondary objectives were to determine the anti-AQP4 IgG status and the length of longest contiguous spinal cord lesion in MS patients. The first study assessing the relation between body weight and MS was published in 1998, wherein an inverse association was observed between high body mass index (BMI) and the risk of MS in Canadian population. [20] The shortcomings of this study were that sample size in this case-control study was small, and BMI was self-reported at diagnosis. It is noteworthy that this study assessed the relationship of BMI with lifetime risk of MS, and not the conversion from CIS to MS. No further study was published in this regard until the start of our study (November 2008) and the impact of BMI on conversion from CIS to MS was not assessed so far in the Taiwanese population. Hence the authors decided to include, amongst other baseline characteristics, the association between baseline BMI and progression from CIS to MS and evaluate its role as a prognostic marker.

## Materials and methods

### Study design

The MS Neo Study is a nationwide, multi-centre, prospective, observational study. Between November 2008 and November 2014, 152 patients aged 6 to 60 years, who had a single, first clinical event of CIS within the last 2 years from study entry were included at 5 institutions in Taiwan. The event had to be a new neurological abnormality present for at least 24 hours, either mono- or polysymptomatic, other than paraesthesia or vegetative dysfunction. Subjects with the diagnosis of MS, or with other disease that could better explain the subject's signs and symptoms, who are scheduled to participate in any interventional treatment trial or who have any condition that could interfere with MRI evaluation, were excluded. For NMO, the revised diagnostic criteria proposed by Wingerchuk DM et al. [18] in 2006 was considered. It required optic neuritis, myelitis, and at least two of three supportive criteria: MRI evidence of a contiguous spinal cord lesion 3 or more segments in length, onset brain MRI nondiagnostic for multiple sclerosis, or NMO-IgG seropositivity.

The study was approved by the institutional review board and ethics committee of each institute and performed in accordance with the principles of the Declaration of Helsinki. The ethics committees that approved our study were China Medical University Hospital Ethics Committee, Chang Gung Memorial Hospital Ethics Committee, Taipei Veterans General Hospital Ethics Committee, National Taiwan University Hospital Ethics Committee, and Kaohsiung Medical University Hospital Ethics Committee. Written informed consent was obtained from all patients.

The Investigators assessed the patient’s disease status periodically until MS or diagnosis with other disease, withdrawal from the study or lost to follow-up. After the baseline visit, the second visit was at 12 to 16 weeks interval, after which periodical telephonic follow-up was done quarterly regarding neurological and visual status. If the patient returned with symptoms or relapse, the ophthalmological and neurological assessment, EDSS and anti-AQP4 IgG status was checked.

33 patients were lost to follow-up and 16 patients did not complete the study. 103 patients completed the study. The study participants had a minimum life expectancy of ≥12 weeks.

According to NHIA regulation, any patients with CIS was not subject to any therapeutic intervention, rather a ‘wait-and-watch’ approach was undertaken.

The authors chose the cut off for EDSS as 0–4.5 and 5–10. Patients having EDSS of 0–4.5 at the end of the study period, were considered to have a mild course of MS; in contrast, patients having EDSS of 5–10 at the end of the study period, were considered to have a moderate to severe course of MS.

### Data collection

During the baseline and the follow up visits, the neurological disability was clinically assessed by the Expanded Disability Status Scale (EDSS). In all patients, baseline and follow-up MRI scans were performed. MRI study included axial and sagittal images of the brain and spinal cord obtained by T1, T2, FLAIR and T1 post-contrast sequences.

The serum AQP4 IgG antibody titre was determined at the baseline visit, follow up visits and at the completion of the study. It was detected using an enzyme-linked immunosorbent assay (ELISA) system according to the manufacturer's instructions (RSR/Kronus, Cardiff, UK) and a level greater than 5 U/mL was considered sero-positive. At the time of study completion, the type of diagnosed disease was determined along with ophthalmological and neurological examination. The date of MS conversion was noted.

The patients could not be screened for anti-MOG-antibodies additionally to anti-AQP-4 IgG antibodies. The current study initiated in November 2008 and by then the robust assays recognizing native MOG protein were under development and were not available within the investigators’ resources. Moreover, the role of anti-MOG antibodies was under investigation. In 2003, Berger T et al. established in a prospective study of 103 CIS patients that analysis of anti-MOG-antibodies in patients with a CIS could predict conversion to MS. [21] These results were soon countered in 2004 by a study conducted by Lampasona V et al. [22] They conducted binding competition experiments using a liquid-phase radiobinding assay to measure serum anti-MOG antibodies in 87 patients with MS, in 12 patients with encephalomyelitis, and in 47 healthy subjects. The study demonstrated that the frequency of positive samples with low titers of anti-MOG IgG (< or = 5.7%) and IgM (< or = 8.3%) was similar in all the groups and subgroups, that these antibodies had low affinity and are not disease specific. Further, in 2005, Lim ET et al. [23] investigated the compatibility of presence of anti-MOG antibody with clinical and MRI demyelination assessments in 47 CIS subjects. The study failed to establish correlation between anti-MOG antibody with MS status based on either the McDonald or Poser criteria. Amidst this rise and fall of anti-MOG antibody, technical challenges of MOG antibody detection were also revealed. It was discovered that Western blot and ELISA tests rather identify antibodies to denaturated MOG whereas the detection of antibodies to correctly folded MOG protein requires advanced methodology such as cell-based assays or tetramer technology. [24, 25]

### Study endpoint

MS was defined by the McDonald criteria 2005 since the study duration was November 2008 to November 2014. The variables analysed were ‘proportion of patients converting to MS or NMO after first episode of CIS’, ‘duration between first episode of neurological event and diagnosis of MS’, ‘anti-AQP4 IgG status’ and ‘length of longest contiguous spinal cord lesion in MS patients. Association between baseline characteristics and progression to MS from CIS was analyzed using multiple logistic regression. Multivariate time dependent effect of baseline characteristics on progression to MS was plotted.

### Statistical considerations

All patients enrolled were included in the analysis. The primary endpoint was summarized as qualitative variable and presented with number of subjects, and percentage. Multiple logistic regression was used to determine the association between baseline demographics/disease characteristics and progression to MS from CIS. A probability of p ≤0.05 was accepted as significant. Kaplan-Meier graph was plotted using duration between initial CIS to MS diagnosis. A forest plot of multivariate time dependent effect of baseline characteristics on the progression to MS was plotted. All analyses were performed using Statistical Analysis Software (SAS) version 9.3.

## Results

### Epidemiological and clinical data

**Fig 1:**
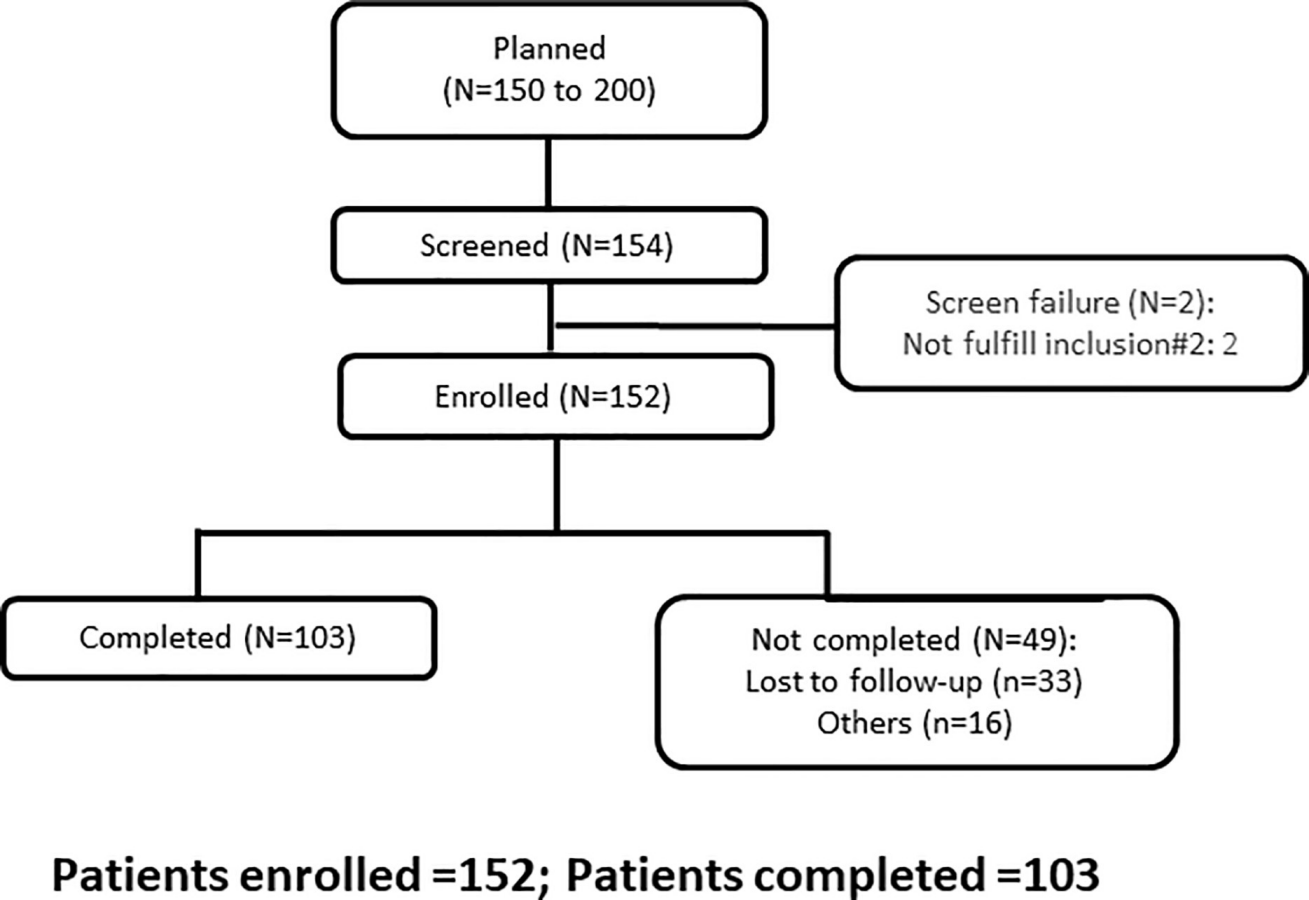
Consort diagram of patient flow.

A total of 152 CIS patients were included in the study and analysed. The study duration was between 4^th^November 2008 (first patient in) to 20^th^ November 2014 (last patient out). The flowchart of patient selection is depicted in Fig 1. All patients enrolled were included in the statistical analysis.

Among the 152 CIS patients included in the study, 116 (76.3%) were women and the mean age at baseline was 38.6 ± 13.3 years (interquartile range, 27.2–50.2). The demographic characteristics of CIS patients at the baseline are shown in Table 1.

**Table 1:** Baseline characteristics of CIS patients.

Parameter	Statistics	Results
Gender	N Female Males	152 116 (76.3%) 36 (23.7%)
Race	N Asians	152 152 (100%)
Age (Year)	N	152
	Mean (STD)	38.6 (13.3)
	Median	38.5
	(Q1, Q3)	(27.2, 50.2)
	(Min, Max)	(9.4, 67.3)
Height (cm)	N	151
	Mean (STD)	160.3 (7.5)
	Median	160.0
	(Q1, Q3)	(155.0, 165.0)
	(Min, Max)	(139.0, 180.0)
Weight (kg)	N	150
	Mean (STD)	58.2 (11.4)
	Median	57.0
	(Q1, Q3)	(50.3, 64.5)
	(Min, Max)	(33.0, 124.0)
BMI (kg/m^2^)	N	150
	Mean (STD)	22.6 (3.7)
	Median	22.3
	(Q1, Q3)	(19.9, 24.8)
	(Min, Max)	(15.1, 40.9)

BMI: Body Mass Index. Subject Didn't Collect Weight: 06–012, Subject Didn't Collect Weight and Height: 01–027.

We analysed the association between baseline demographic characteristics (age, gender and BMI) in CIS patients and the conversion rate to MS using multiple logistic regression and did not find any statistically significant association as shown in Table 2. Association between race and MS conversion could not be analyzed as all the enrolled subjects were Asians.

**Table 2:** Association between baseline demographics/disease characteristics and conversion to MS.

Parameter	Category	Coefficient	E^Coefficient (OR)	P-value
**Age (Year)**		–0.016	0.984	0.363
**BMI (kg/m**^**2**^**)**		0.017	1.018	0.778
**Gender**	Male vs. Female	–0.180	0.835	0.757
**Location of Longest Lesion**	Peripheral vs. Central	–1.131	0.323	0.459
	Holospine vs. Central	–13.551	<0.001	0.974
	Central+Peripheral vs. Central	13.683	>999.999	0.990
	NA vs. Central	–2.293	0.101	0.014
**EDSS Score**		0.170	1.186	0.135

Analyzed using multiple logistic regression to evaluate association between baseline demographics/disease characteristics and conversion to MS from CIS.

BMI: Body Mass Index; EDSS: Expanded Disability Status Scale

Amongst all the patients enrolled, 142 (93.4%) presented with CIS at the baseline visit and the remaining 10 patients (6.6%) had presented prior to the baseline visit. The type of episode was optic neuritis (ON) in 72(47.4%) patients and transverse myelitis (TM) in 47(30.9%) patients. The clinical episode in one patient (0.7%) consisted of simultaneous ON and TM. Apart from ON and TM, 32(21.1%) patients experienced other syndromes such as encephalopathy, brainstem encephalitis, right hemispheric attack, and acute disseminated encephalomyelitis (ADEM).

A total of 22 (14.5%) patients converted from CIS to MS, with an average duration of 1.1 ± 1.0 years (interquartile range, 0.2–1.9). 5 (3.3%) patients developed NMO. Thus, conversion rate to collectively either MS or NMO was 17.8% during 4-year observation period. MS developed in 7 out of 72 (9.7%) who initially presented with ON and in 8 out of 47 (17.0%) with a spinal cord syndrome. Six of 32 patients (18.8%) with other syndromes converted to MS and one patient who had presented with simultaneous event of ON and TM also converted to MS.

**Fig 2:**
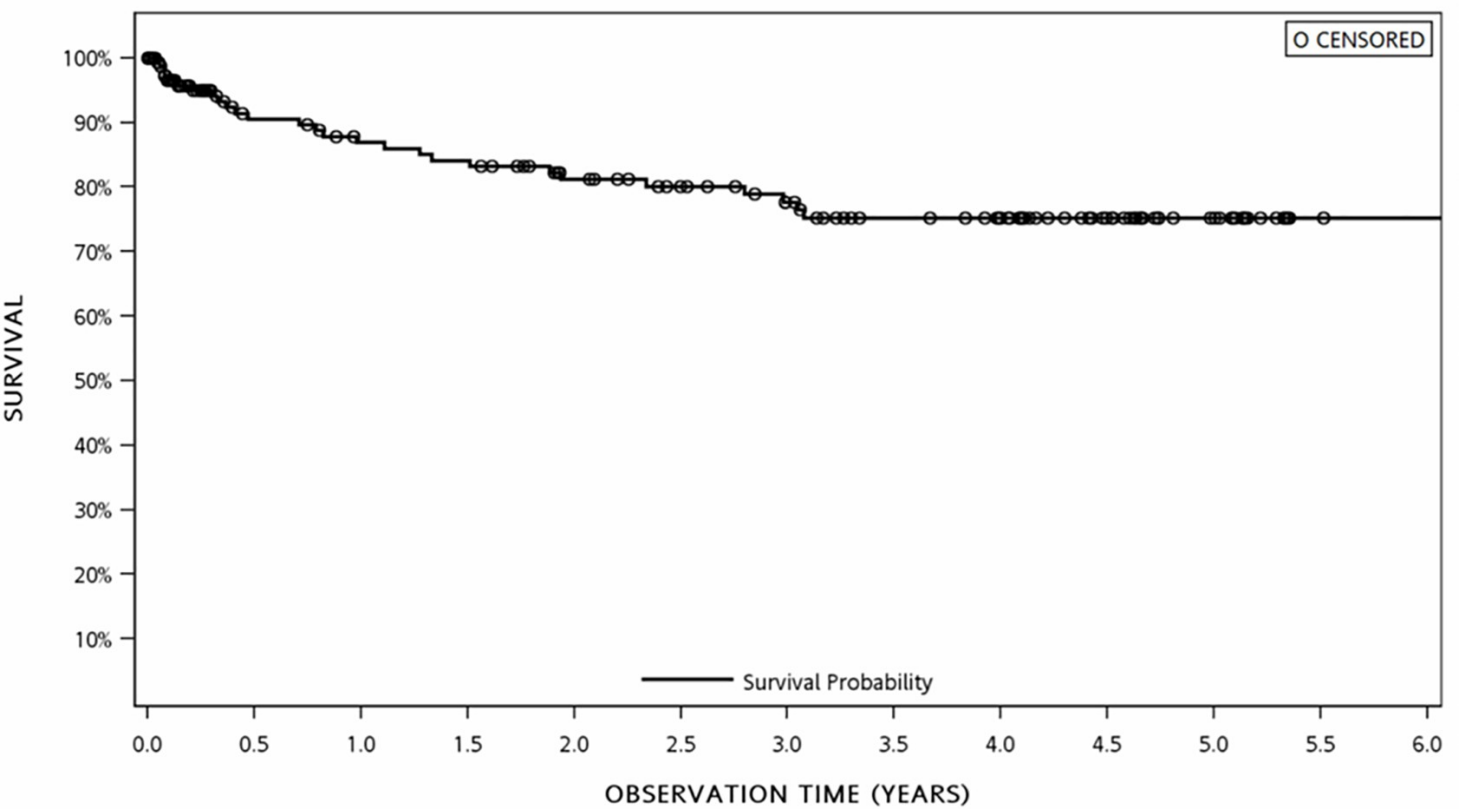
KM plot of duration between initial CIS to MS diagnosis.

Eleven patients (50%) developed MS within six months of first CIS and three (13.6%) converted to MS within a year. The same finding has been depicted in the Kaplan-Meier plot of duration between initial CIS to MS diagnosis. (Fig 2)

The median baseline EDSS score in patients who developed MS was 2.0. The score at the end of follow up was found to be between 0.0 to 4.5 in 8 (61.5%) patients with a mild disease course, and score was between 5.0 to 10.0 in 5 (38.5%) subjects with a moderate to severe course. The median EDSS score in NMO subjects was 3.5 at baseline and 2.5 at the end of follow up period. There was no significant association between the EDSS score and conversion to MS (P = 0.135). [Tables 2 and 3]

**Table 3:** Median EDSS score at baseline and follow-up visits.

Visit	Statistics	All subjects	MS subjects	NMO subjects
**Baseline Visit**	N	152	22	5
	Median	2.0	2.0	3.5
	(Min, Max)	(0.0, 9.0)	(0.0, 9.0)	(2.0, 7.5)
	Between 0.0 to 4.5	140 subjects (92.1%)	20 subjects (90.9%)	3 subjects (60.0%)
	Between 5.0 to 10.0	12 subjects (7.9%)	2 subjects (9.1%)	2 subjects (40.0%)
**Visit 2 (12–16 weeks after baseline)**	N	104	15	3
	Median	1.0	1.0	1.5
	(Min, Max)	(0.0, 7.5)	(0.0, 5.0)	(0.0, 2.0)
	Between 0.0 to 4.5	100 subjects (96.2%)	14 subjects (93.3%)	3 subjects (100%)
	Between 5.0 to 10.0	4 subjects (3.8%)	1 subject (6.7%)	0 subject (0.0%)
**Relapse/** **Symptomatic Visit**	N	26	13	4
	Median	3.0	3.0	2.5
	(Min, Max)	(0.0, 9.0)	(1.0, 9.0)	(1.0, 6.0)
	Between 0.0 to 4.5	19 subjects (73.1%)	8 subjects (61.5%)	3 subjects (75.0%)
	Between 5.0 to 10.0	7 subjects (26.9%)	5 subjects (38.5%)	1 subject (25.0%)

### Neuroimaging study

Seven (46.7%) patients with an initial episode of optic neuritis who converted to MS were having an abnormal brain MRI scan at the baseline. The average number of contrast enhanced lesions in these patients was 0.6 ± 0.5 (interquartile range, 0.0–1.0) and only one patient fulfilled the Barkhoff criteria in the baseline brain MRI.

The spinal MRI revealed that the average number of lesions were 1.2 ± 1.1. The length of the longest spinal cord lesion at baseline was 2.0 ± 0.8 (range 1.0–3.0) in patients who converted to MS and 3.5 ± 0.7 (range 3.0–4.0) in patients who developed NMO. The location of the longest lesion was central in 50% of patients followed by peripheral (25%) and holospine (16.7%). Multiple logistic regression revealed no significant association between the location of the longest lesion in baseline MRI and conversion to MS. (Table 2)

### Anti-AQP4 IgG antibody status

Amongst the patients who developed NMO, 4 of 5 (80.0%) were positive for anti-AQP4 IgG antibody.

**Fig 3:**
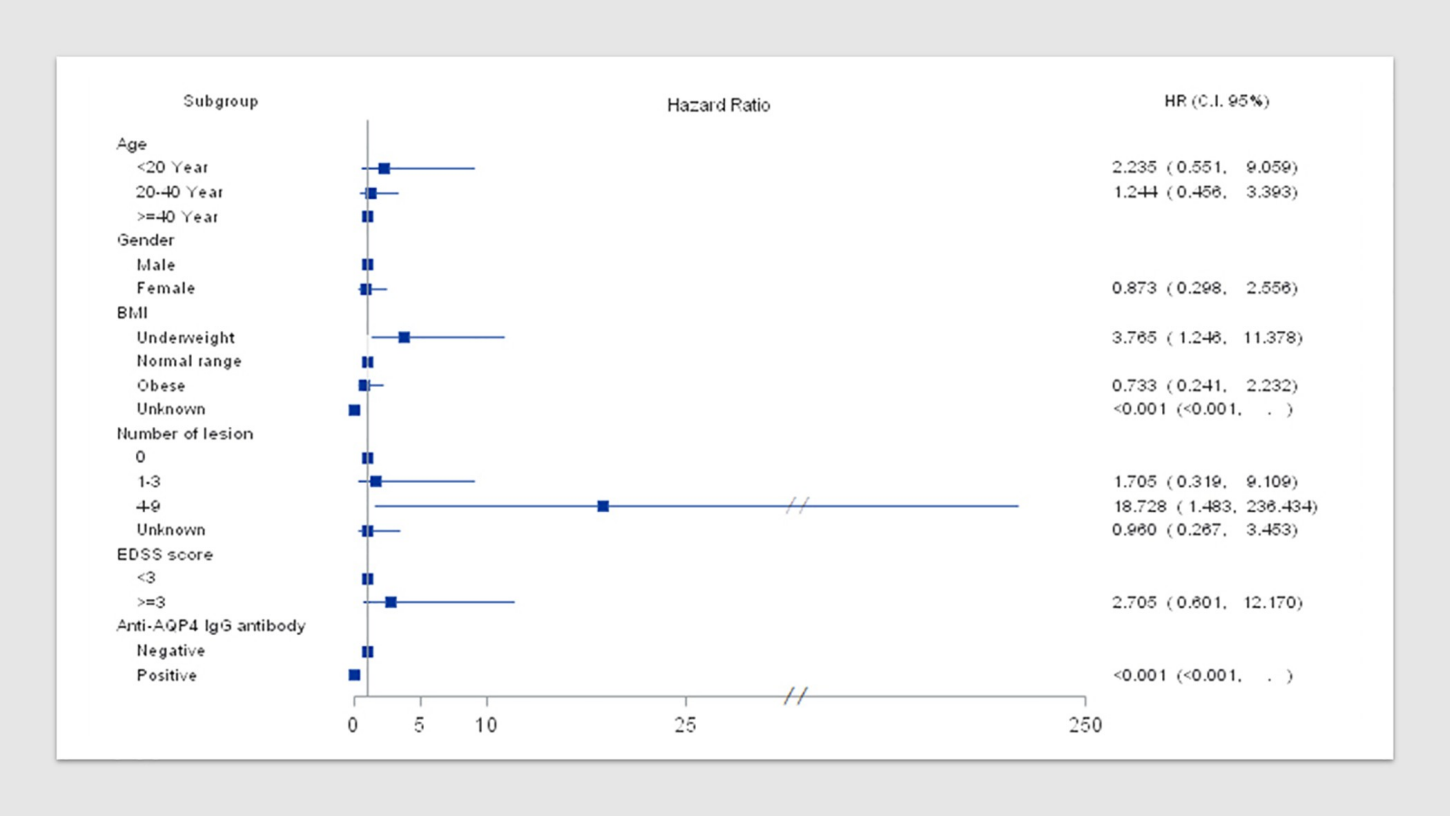
Forest plot of multivariate time dependent effect of baseline demographic/ disease characteristics on the conversion to MS (enrolled population).

A multivariate analysis was conducted incorporating time-dependent effect of demographic and disease characteristics, clinical and radiological data available at baseline. In the multivariate analysis (Fig 3), our study cohort revealed that ‘BMI’ and ‘number of lesions’ had significant association with probability of CIS to MS conversion. ‘Age’, ‘gender’ and ‘EDSS’ had non-significant association with MS conversion rate but a trend was noted.

## Discussion

The present study provides prospectively acquired epidemiological, clinical and MRI data over a period averaging 4 years from CIS onset in Taiwanese population. The overall conversion rate from CIS to MS was 14.5% (22/152). The conversion rates to MS in our study is lower than observed in most of the trials including the optic neuritis treatment trial. (Table 4).

**Table 4:** Previous studies of the conversion rate of multiple sclerosis following idiopathic optic neuritis.

Study	Year	No. of patients (n)	Conversion rate	Follow up period
Optic neuritis study group (USA) [3]	1997	389	50%	15 years
Fisniku et al. (UK) [11]	2008	54	65%	20 years
Kuhle et al. (UK) [4]	2015	1027	59.5%	4.31 years
ZhonghuaNeiKe (China) [26]	2016	151	30.5%	44.11±17.62 months
Saxena et al. (India) [27]	2016	83	28%	18 months
Marques et al. (Portugal) [28]	2014	42	23.8%	5 year
Isayama Y et al. (Japan) [29]	1982	-	8.3%	-
Corona-Vazquez T et al. (Mexico) [30]	2009	110	12%	7 years

The finding of overall low conversion rate to MS of Taiwanese patients complement the results of the earlier retrospective studies which reveals low risk of MS in Taiwan [2,5]. The variability in conversion rates (ranging between 8.3% to 65%) due to geographic, genetic and ethnic factors, and also due to differences in study design, frequency and duration of follow up, sample size and method of assessment is observed. Standards for conducting long-term studies need to be established.

The conversion rates of ON to MS in our study was 9.7% (7/72). It similar to two studies conducted in Japan and Mexico (Table 4). In Japan, a statistical survey of ON revealed that rate of conversion of ON to MS was 8.3% [29]. A partially retrospective and longitudinal study in Mexico showed that the percentage of patients that converted from primary idiopathic ON to a clinically definite MS in a follow-up period of 7 years was 12% [30]. It suggests that conversion rates of ON to MS does not vary greatly across ethnicities.

### Gender predilection

Among all the patients who converted to MS, a female preponderance was seen in the ratio of 3:1. This finding is consistent with the well described gender predilection of MS [1,2,3,6,31].

### Age of onset

The mean age at the onset of neurological symptoms (38.6 years) in our study is similar to the mean age reported by the ONTT and other major trials [3,4,11,27,28,31]. However, contrary to other studies, we did not find any association between mean age at onset and gender with the risk to MS conversion [3,10].

### Duration until MS conversion

Our study reported that half of the patients (11 patients) developed MS within six months of first CIS. Analogous to this study, Kuhle *et al*., in a large international cohort, found the duration to conversion was in the range of 7 months to 2 years [4]. This probably implies the impact of larger sample size of the results. Marques *et al*. reported that majority of ON patients converted to MS during the first follow-up year [28].

### Prognostic factors for CIS to MS conversion

A multivariate analysis was conducted incorporating time-dependent effect of demographic and disease characteristics on the conversion to MS. (Fig 3) Greater risk of relapse in younger patients in the age group of < 20 years was observed compared to patients aged ≥ 40 years at disease onset, however, the statistical significance of this result could not be proven. Gender does not seem to be a risk factor for conversion to MS or a further relapse, in line with the gender predilection stated in literature [32,33]. Nonetheless, we cannot exclude the probability that a longer follow-up period might demonstrate a difference in MS conversion among genders. Similar to our study, another study by Tintore M et al. [34] reported lack of gender predilection for conversion rate from CIS to MS in multivariate analysis with an observation period of 18 years. This again highlights the impact of ethnicity and race on the natural course of CIS.

Our study revealed significant association between the baseline BMI and conversion to MS. It showed that being underweight is a significant risk factor for conversion from CIS to MS. This is the first study to report an inverse association with BMI and risk of conversion from CIS to MS. Our results are partly echoed by a Canadian case control study of 197 incident cases and 202 frequency matched controls which employed a 164-item food frequency questionnaire in a face-to-face interview. An inverse association was observed between high BMI and the risk of MS, with an odds ratio (OR) of 0.76 (95% confidence interval [CI]: 0.61–0.95), per 5-unit increase in BMI, both sexes combined. Thus, a 5-unit increase in BMI was associated with a 31% reduction in MS risk. But this study did not examine the association between CIS to MS conversion, instead association of ‘chances of occurrence of MS’ with BMI.[20] On similar lines, Langford A et al. conducted a retrospective cohort study of 305 incident CIS cases between 2008 and 2009 with an average duration of follow up of 3.5 years. They found that obesity at the time of symptom onset was not associated with an increased risk of conversion to MS in either univariate or multivariate analyses. [35] A possible explanation of our results could be that most of the studies which have established a direct relationship between BMI and propensity for MS, have recorded the patients self-reported BMI in the age group of 18–25, whereas our study recorded baseline BMI at initiation of this study (median age 38.5; range 9.4–67.3). [36–38] Another reason could be due to the low sample size of this study. Nevertheless, the role of BMI as a prognostic marker is subject to further larger trials in Taiwanese cohort. Obesity can impact the natural course of MS in two ways. Elevated levels of serum 25-hydroxyvitamin D appear to decrease MS risk. Obese individuals have lower levels of vitamin D metabolites, including 25-hydroxyvitamin D, than normal weight individuals, and therefore obesity could be an important risk factor for MS. Further, obesity is associated with a low-grade chronic inflammatory state and release of cytokines that affect immune responses and possibly MS risk.

Though some studies have shown that ON is associated with a lower risk of MS, the risk is comparable across different CIS subtypes [1]. In this report, CIS patients presenting with other syndromes had greater predisposition to convert than the patients with ON and/or TM at the time of presentation. These findings may be attributable to differences in patient inclusion criteria, diagnostic criteria for MS and the duration of the follow-up period in various studies.

The group which converted to MS in our study exhibited a narrow spectrum of extent of disability. Whilst 91% had a mild course with minimal disability (EDSS < 4.5), only a few patients (9%) developed increased neurological disability (EDSS 5–10). The current definition of SPMS states absence of a relapse, confirmed after 3 months within the leading Functional System and an EDSS step ≥4 and pyramidal score ≥2. The knowledge is insufficient to categorize these patients as SPMS. Hence, we consider them as highly impaired RRMS or patients with a severe course and greater disability. The percentage of people with a mild course of multiple sclerosis in our cohort was higher than reported on other cohort of patients. In a study with a follow up period of 20 years, 42% of patients had substantial neurologic disability with a median EDSS score of 6.5 [11].

In our study, 80% patients who developed NMO were tested positive for anti-AQP4 IgG antibody and 4.7% patients fulfilled the criteria for NMOSD suggestive of a high risk of future relapses [16].

The analysis confirms that MRI at baseline is a robust factor predictive of conversion to multiple sclerosis. Almost half of the patients who converted to MS after 4 years had demyelinating lesions of the brain and spinal cord on MRI scan at baseline with Gd contrast enhanced lesions. The number of lesions ranging from 4 to 9 on MRI scan at baseline is a significant risk factor for conversion to MS which is consistent with the findings by Tintore M et al. [34] The duration to convert to MS from ON is generally associated with the number and location of demyelinating lesions of the brain and spinal cord [2,10]. In this study, there was a clear distinction in the conversion rates between patients with and without evidence of prior demyelination on brain MRI. In keeping with several previous studies, we confirmed the fact that abnormal baseline brain MRI is a strong predictor for the development of MS in idiopathic ON patients [3,4,11].

Further, an important clinical factor is the extent of neurological disability assessed by EDSS score. Our study showed that patients with an EDSS score ≥ 3 display a higher risk of conversion to MS. Patients who tested positive for anti-AQP4 IgG antibody had NMO as diagnosed by modern criteria.

### Clinical relevance of researching for prognostic factors

Currently available treatments have unequivocal efficiency and may slow the conversion to relapsing-remitting MS but could be avoided for patients with mild disease course [39]. Niino M et al. in their review have weighed the findings from multiple studies and concluded that an initial approach of wait-and-see for the use of disease modifying treatment would serve the best interests of patients who experience a clinical episode of mild and reversible CIS, even if accompanied by an abnormal scan.[40] We did not perform any therapeutic intervention according to NHIA regulation, rather a ‘wait-and-watch’ approach was undertaken.

### Strengths and limitations

A key strength of our study was inclusion of patients from majority of medical centres in the country. However, the study had some limitations, including a relatively small sample size. We could not assess the vitamin D levels in this study. Also, we could not determine the effect of ethnicity on the clinical course as our study included only Asian population. Furthermore, the variables assessed were not adjusted for ancestry and genetic variants with an inherent MS risk, thereby limiting the generalization of our results. EDSS scale used has inherent limitations like intra-observer variability and heavy weightage given to physical disability with limited assessment of key clinical features. Furthermore, MS conversion rate may have been underestimated as only clinical MS conversion was considered and not the new silent subclinical lesions.
